# Supplementary material for: Haisu: Hierarchically supervised nonlinear dimensionality reduction
Source: PLoS Comput Biol. 2022 Jul 21;18(7):e1010351. doi: 10.1371/journal.pcbi.1010351 (PMC9345488; doi:10.1371/journal.pcbi.1010351)
Supplement: S1 Text — Table A in S1 Text: Mean runtimes (mm:ss.s) correspond to Fig 2,3,4 for the Haisu hierarchy-base pairwise distance matrix and downstream choice of NLDR method. Each method was run with no subsampling across 48 cores and respective parallel program parameters. T-SNE, UMAP, PHATE, and PCA runtimes do not include the pair-wise distance calculation time and (other than PCA) are run with the ‘precomputed’ metric parameter*. Thus, the runtime of Haisu is contingent on the size of the input dataset and the accompanying metric used. We have provided parallel and optimized single-thread versions to facilitate processing large datasets. Table B in S1 Text: Haisu better represents a hierarchy graph in an embedding when compared to equal-weighted, independent classes. We take the area under each graph (AUC) integration using Simpson’s rule. Scores are averaged for strength factors from 0 to 0.999 or integrated by a step size of 0.1. We include HS* = 1-HS such that higher scores (bolded) are more favorable, indicating that Haisu more accurately represents the hierarchy graph while maintaining integrity of the embedding. Table C in S1 Text: Haisu better represents a hierarchy graph in an embedding when compared to equal-weighted, independent classes. We compare methods by nearest neighbor distribution similarity with the input hierarchy graph (HC) and by a hierarchically modified silhouette score (HS). For every dataset and scoring metric, Haisu demonstrates significant benefits for representing the input hierarchy as compared with equally weighted classes. HC* = 1‐(HC) and HS* = 1-HS such that higher values of HC* and HS* are favorable. Table D in S1 Text: No NLDR method is consistently more compatible with Haisu. We compare methods by nearest neighbor distribution similarity with the input hierarchy graph (HC) and by a silhouette score (HS). HC is calculated across KNN = 3, n/100, n/10 where n is the total number of samples for a given dataset and KNN is the k-nearest neighbors [file pcbi.1010351.s001.docx]

**Supplementary Information**

| **Dataset** | **Haisu** | **t-SNE*** | **UMAP*** | **PHATE*** | **PCA*** |
| --- | --- | --- | --- | --- | --- |
| PBMC | 05:44.60 | 04:38.53 | 03:11.31 | 15:39.14 | 01:26.10 |
| Epithelial | 04:25.30 | 04:11.24 | 02:33.84 | 13:12.58 | 01:10.17 |
| Cardiac | 00:02.15 | 00:03.04 | 00:10.09 | 00:02.55 | 00:00.03 |

***Table A:*** *Mean runtimes (mm:ss.s) correspond to Fig. 2,3,4 for the Haisu hierarchy-base pairwise distance matrix and downstream choice of NLDR method. Each method was run with no subsampling across 48 cores and respective parallel program parameters. T-SNE, UMAP, PHATE, and PCA runtimes do not include the pair-wise distance calculation time and (other than PCA) are run with the ‘precomputed’ metric parameter*. Thus, the runtime of Haisu is contingent on the size of the input dataset and the accompanying metric used. We have provided parallel and optimized single-thread versions to facilitate processing large datasets.*


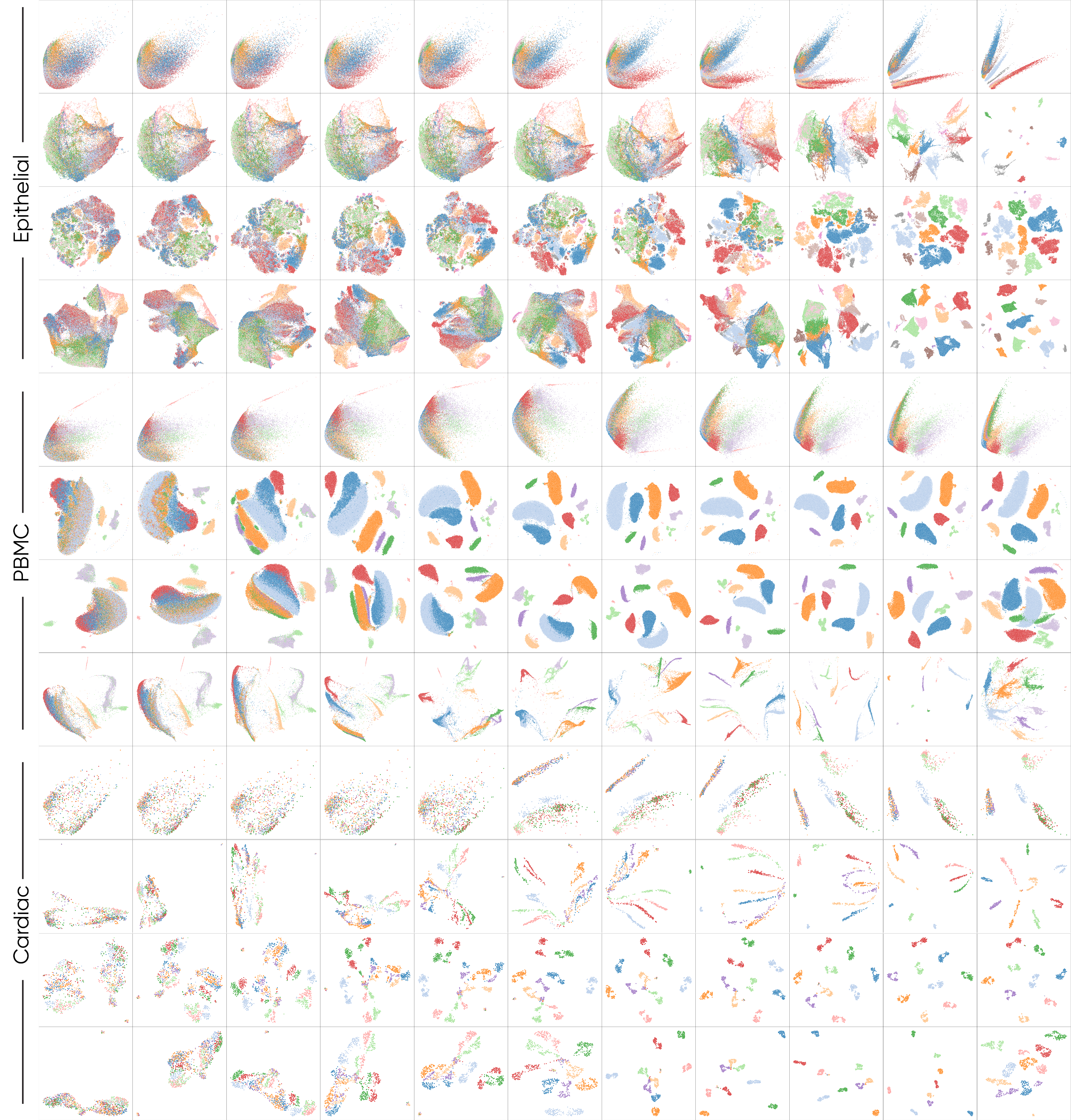


***Fig. A:*** *We display the full progression of Haisu from a strength factor of str=0 to str=0.999 by a step size of 0.1 corresponding to Fig. 2,3,4. Modified PCA, PHATE, t-SNE, and UMAP are displayed from top to bottom for each dataset.*


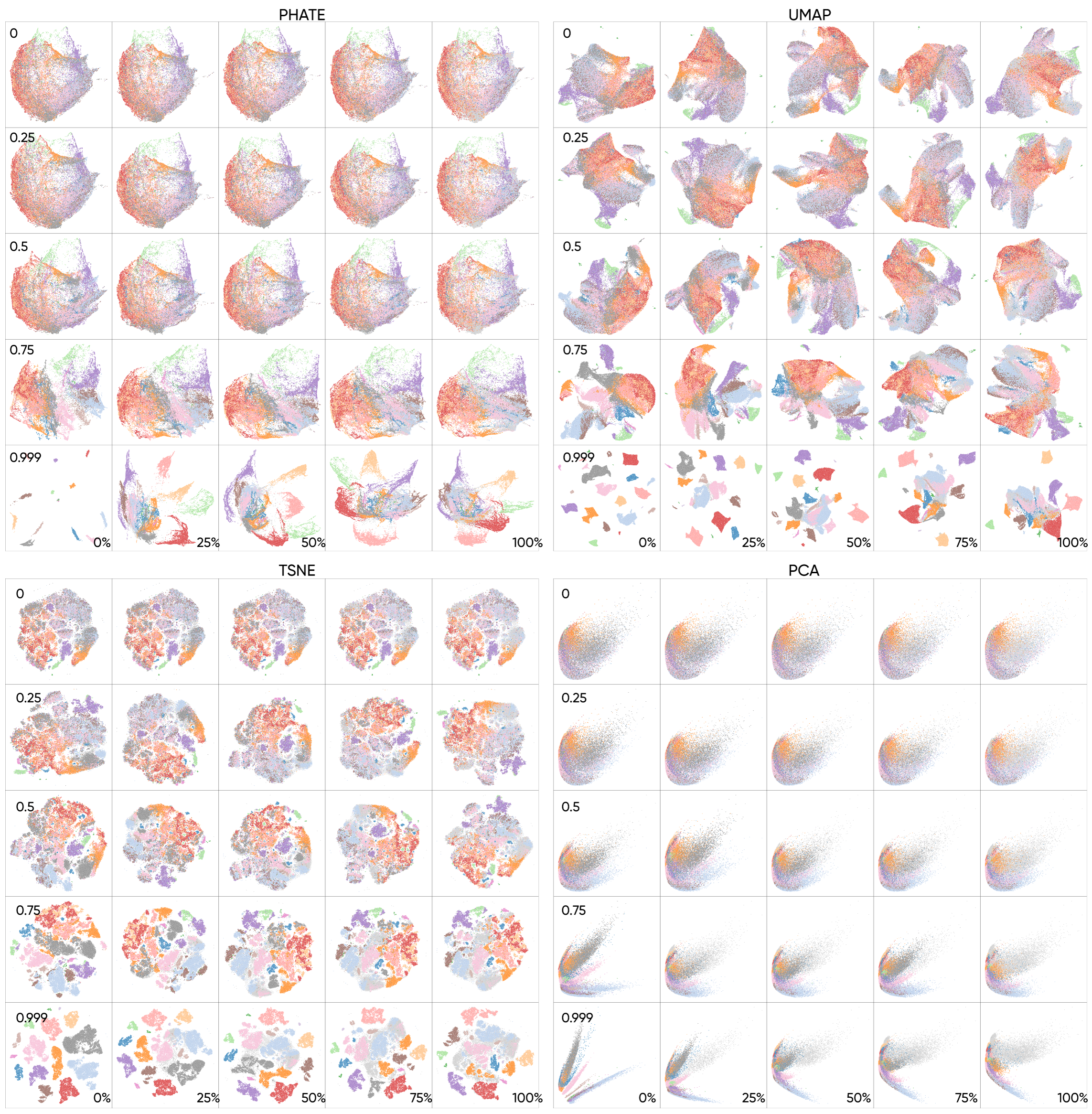


***Fig. B:*** *Full progression of Haisu strength factor values and percentage removal of the TA-1 label corresponding to Fig. 5. Modified PHATE, t-SNE, PCA, and UMAP nonlinear dimensionality reduction techniques are depicted. The y-axis indicates str value and the x-axis indicate percentage removal of the TA 1 label.*

**Supplementary Note 1**

*Choice of Haisu Strength Factor*

*
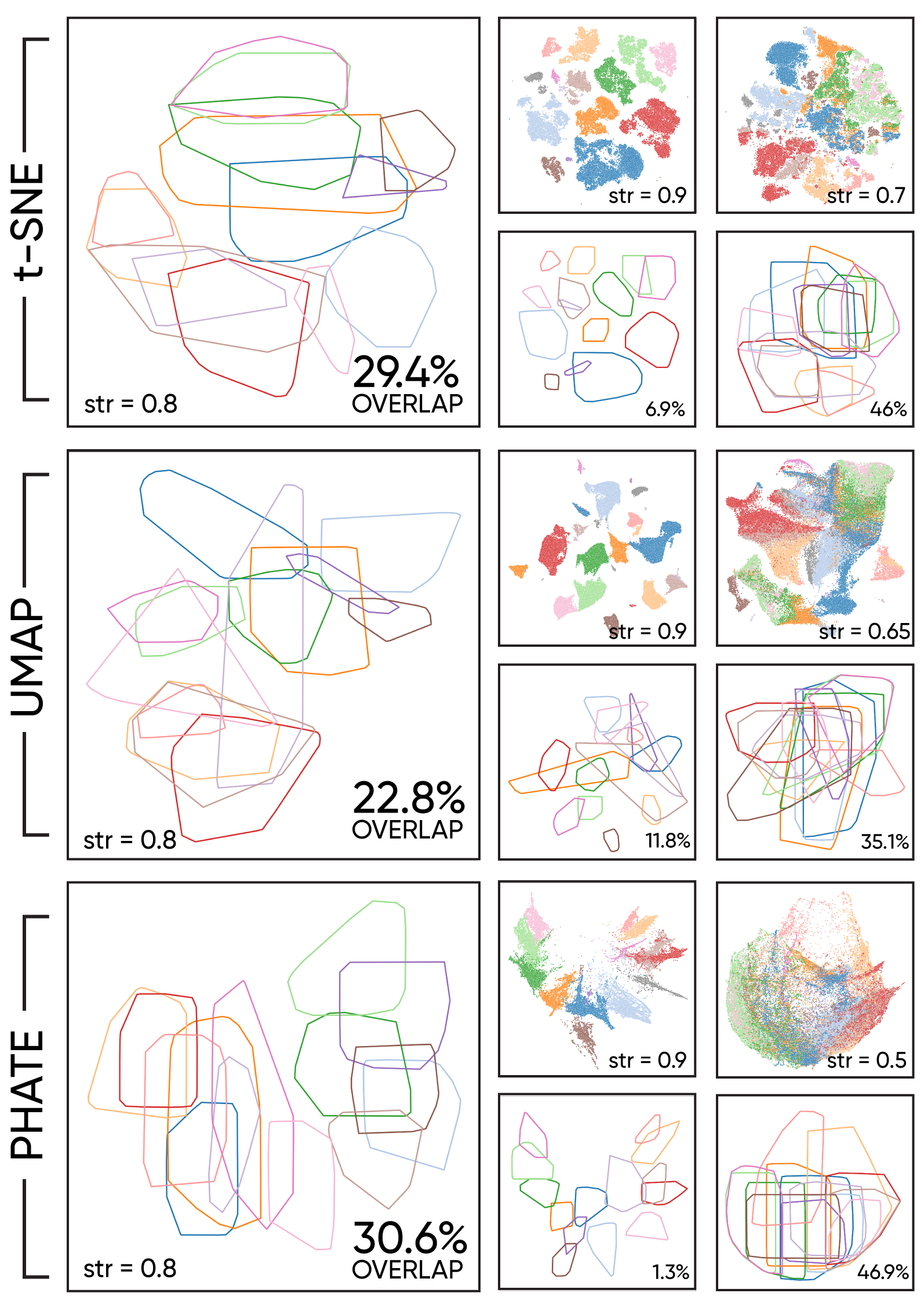
*

***Fig. C****: We demonstrate a heuristic for aiding in the choice of hierarchical distancing factor (str). For the epithelial dataset (shown), we favor overlap scores between 35% and 45%, which visually demonstrate hierarchically influenced cluster separation while maintaining characteristics of the raw embedding. To find mean overlap, we compute the nearest shape intersection for each cluster’s convex hull. Clusters are determined by the input classes and outliers are first removed by z-score.*

In Fig. C we illustrate an example visual method with which to select a strength value for modulating the effect of Haisu’s hierarchical distancing factor. For each set of points in the embedding, grouped by class label, we calculate the convex hull after removing outliers with z-score / 4. We then find the nearest shape by centroid for each convex hull and average the area intersections. Due to its use of z-score, this technique is designed for starting from high strength values and decreasing until the desired overlap is found.

To help facilitate use of this technique, we have provided an auto-runner that searches for 50% class overlap over a maximum number of iterations for which to test strength values. We provide this program as a modifiable guide to aid in choice and understanding of strength values for a given input hierarchy. In our source repository <https://github.com/Cobanoglu-Lab/Haisu> we provide access to this auto-runner.

*
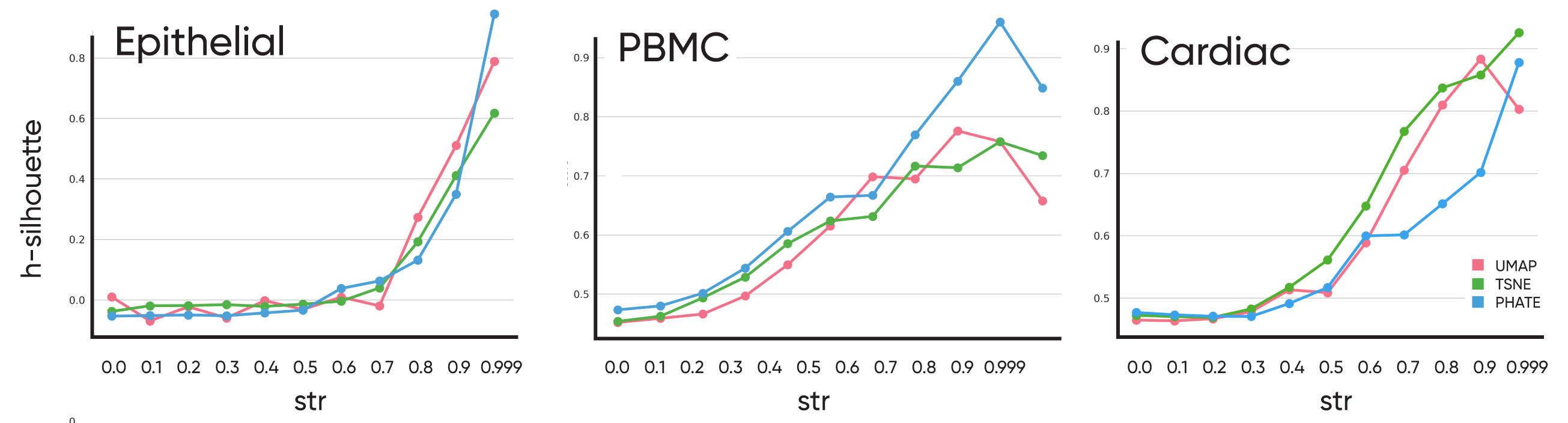
*

***Fig D: The h-silhouette score can be used to find an ideal range of Haisu strength values.*** *We demonstrate a hierarchically modified silhouette score (h-silhouette) that determines how effective a given strength value is at maintaining inter-class distance in the embedding, weighted by the input hierarchy. A score closer to zero is more desirable.*

For a more grounded analysis of what determines an ideal strength value for a given dataset we explore a hierarchically modified silhouette score in Fig. F. This score (*HS*) is determined by:

$$HS = \left( \theta_{ij}\cdot b_{j}-a_{i}/maxdist \right)/\max\left( a_{i}/maxdist,\theta_{ij}\cdot b_{j} \right)$$

Here *a* is the mean intra-cluster distance for points of class *i*, and *b* is the distance to the nearest cluster of class *j.* Thus, a lower *HS* is desirable because closer clusters in the embedding that are also closer in the hierarchy graph will favor lower values. For the datasets in this text, where NLDR parameters have already been tuned, we found an optimal strength value to lie at ${HS < HS}_{0}+0.1$ for maximizing hierarchy quality while minimizing dramatic distancing between clusters where ${HS}_{0}$ is the *HS* score for a strength value of zero.

In this manner, a user can inspect the h-silhouette score (*HS*) to target an embedding where the Haisu does not dramatically distance clusters while still achieving a significant amount of hierarchical perturbation. If preservation of local inter-class relationships are favored, a user will want to choose the furthest right *HS* value that is not dramatically different from the *HS* value at *str=0*. If hierarchy integrity is a priority, then the furthest left values that are dramatically different from the *HS* value at *str=0* are more favorable. In Fig. D we observe that our desired strength values for each dataset occur when the graph starts to increase +0.1 or +0.2 strength. It is important to note that choice of strength value is highly dependent on the parameters of the integrated NLDR approach, thus recommendations for *str* are entirely dependent on a user’s desired degree of class separation in the graph and choice of parameters. At higher strength values, graph parameters (ex. perplexity) will need to be adjusted to compensate for denser clusters.

**Supplementary Note 2**

*Hierarchy Metrics for Class Independence Testing*

Here we investigate the extent of Haisu’s hierarchical modification on a given embedding. To support the efficacy of the input hierarchy on the resulting embedding, we demonstrate that Haisu is more effective at preserving graph relationships than a paradigm where each class is equidistant in the input graph.

| **Dataset** | **Method** | **HS*** | **HS* AUC** |
| --- | --- | --- | --- |
| Cardiac | Equal | 0.339 | 1.029 |
|  | **Haisu** | **0.393** | **1.300** |
| PBMC | Equal | 0.250 | 0.795 |
|  | **Haisu** | **0.373** | **1.205** |
| Epi | Equal | 0.676 | 2.210 |
|  | **Haisu** | **0.886** | **2.819** |

***Table B: Haisu better represents a hierarchy graph in an embedding when compared to equal-weighted, independent classes.*** *We take the area under each graph (AUC) integration using Simpson’s rule. Scores are averaged for strength factors from 0 to 0.999 or integrated by a step size of 0.1. We include HS* = 1-HS such that higher scores (bolded) are more favorable, indicating that Haisu more accurately represents the hierarchy graph while maintaining integrity of the embedding.*

Foremost, we integrate the h-silhouette score introduced in Supplementary Note 1, to immediately demonstrate that for all graphs, Haisu has a lower (more favorable) HS value when the input hierarchy is used as compared to the case where nodes are equidistant in the graph. In Table B, we also include the area under each resulting distribution of *HS* values to better represent positive and negative changes between strength values of the same method because we are averaging PHATE, t-SNE, and UMAP *HS* scores.

To supplement *HS*, we also provide a metric that compares neighborhood distributions in an embedding with an expected graph distribution formulated from the hierarchy. Let *n* be the total number of samples, *u* be the number of unique labels, and $\mathcal{l}_{i}$ be the number of samples with an indexed label *i*. The expected set of frequencies *e* is formulated as:

$$e_{ij}=\theta_{ij}\cdot\mathcal{l}_{j}$$

$$e_{i} = \{e_{i1}, e_{i2}, ..., e_{iu}\}/\sum_{j} e_{ij}$$

For a given sample $x_{i}$ we then compare the expected frequencies $e_{i}$ with the *l-1* normalized labels counts from the *k-*nearest neighbors of $x_{i}$ to obtain $o_{i}$. With observed values $o_{i}$ and expected values $e_{i}$ we compute the modified chi-squared statistic $HS=\mathcal{X}^{2}$.

*
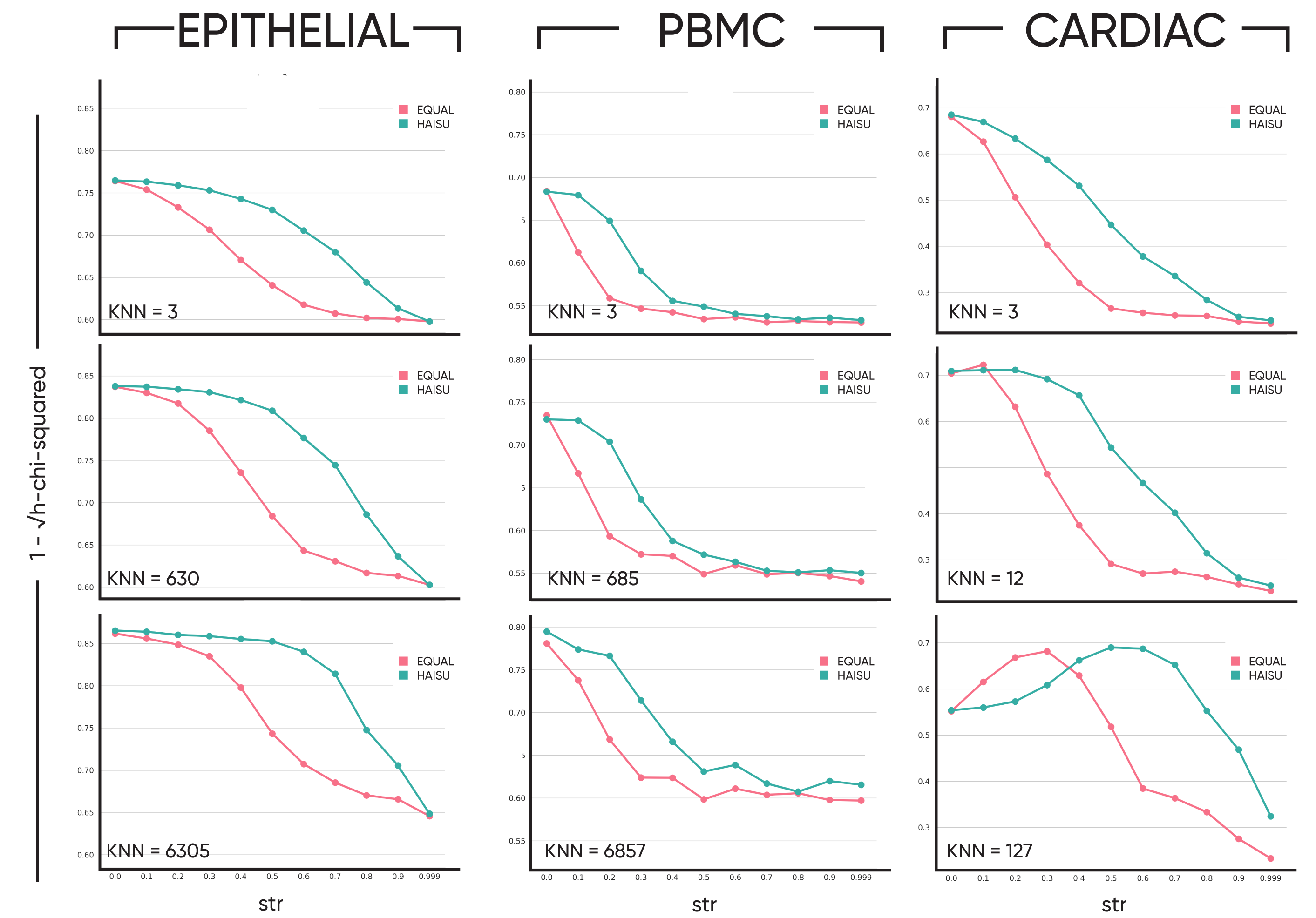
*

***Fig. E:*** ***Haisu better represents the expected hierarchy labels in local and global neighborhoods when compared to equal-weighted, independent classes.*** *We calculate the modified “HC” score for each dataset across multiple choices of k-nearest neighbors (KNN). HC is determined by calculating the mean chi-squared statistic for the observed v.s. expected distribution of k labels surrounding each point in the embedding. We report 1 minus the square root of the resulting score (y-axis) s.t. values closer to 1 are more desirable. The expected distribution reflects the input hierarchy graph l-1 normalized from 1/maxdist to 1. HC is calculated across KNN = 3, n/100, n/10 where n is the total number of samples for a given dataset and KNN is the k-nearest neighbors including self.*

| **Dataset** | **Method** | **HC* AUC** | **HC*** | **HS*** | **HS* AUC** |
| --- | --- | --- | --- | --- | --- |
| Cardiac | Equal | 4.126 | 0.417 | 0.339 | 1.029 |
|  | **Haisu** | **5.157** | **0.517** | **0.393** | **1.300** |
| PBMC | Equal | 11.500 | 0.584 | 0.250 | 0.795 |
|  | **Haisu** | **12.046** | **0.611** | **0.373** | **1.205** |
| Epi | Equal | 13.794 | 0.700 | 0.676 | 2.210 |
|  | **Haisu** | **14.809** | **0.752** | **0.886** | **2.819** |

***Table C: Haisu better represents a hierarchy graph in an embedding when compared to equal-weighted, independent classes.*** *We compare methods by nearest neighbor distribution similarity with the input hierarchy graph (HC) and by a hierarchically modified silhouette score (HS). For every dataset and scoring metric, Haisu demonstrates significant benefits for representing the input hierarchy as compared with equally weighted classes. HC* = 1-*$\sqrt{\left( HC \right)}$ *and HS* = 1-HS such that higher values of HC* and HS* are favorable.*

Using HC as hierarchically modified chi-square statistic, we compare the unmodified Haisu graph with the equidistant label graphs “Equal” in Fig. E. Results are compiled for local (k=3), large (k=n/100), and global (k=n/10) neighborhoods. We observe that using the original hierarchy through Haisu is significantly more effective for *HC* in local and large neighborhoods. For *HC* and *HS,* Haisu outperforms the equidistant labeling scheme across all datasets and methods (Table C). Thus, we support via two metrics that Haisu is more effective at preserving graph relationships than a paradigm where each class is equidistant in the input graph.

**Supplementary Note 3**

*Comparison of NLDR Methods*

| ***Dataset*** | ***Method*** | **HC* AUC** | **HC*** | **HS*** | **HS* AUC** |
| --- | --- | --- | --- | --- | --- |
| Cardiac | PHATE | **1.707** | 0.533 | **0.424** | **0.427** |
|  | t-SNE | 1.606 | 0.472 | 0.363 | 0.408 |
|  | UMAP | 1.657 | **0.548** | 0.392 | 0.390 |
| PBMC | PHATE | **3.995** | 0.614 | 0.330 | 0.313 |
|  | t-SNE | 3.961 | 0.603 | 0.391 | 0.349 |
|  | UMAP | 3.972 | **0.616** | **0.398** | **0.360** |
| Epi | PHATE | **3.995** | **0.767** | 0.887 | 0.866 |
|  | t-SNE | 3.961 | 0.733 | **0.898** | **0.879** |
|  | UMAP | 3.972 | 0.756 | 0.874 | 0.865 |

***Table D:*** ***No NLDR method is consistently more compatible with Haisu.*** *We compare methods by nearest neighbor distribution similarity with the input hierarchy graph (HC) and by a silhouette score (HS). HC is calculated across KNN = 3, n/100, n/10 where n is the total number of samples for a given dataset and KNN is the k-nearest neighbors including self. Graphs are calculated for strength factors from 0 to 0.999 by a step size of 0.1. We take the area under each graph (AUC) integration using Simpson’s rule. HC* = 1-*$\sqrt{\left( HC \right)}$ *and HS* = 1-HS such that higher values of HC* and HS* are favorable.*
