# Supplementary material for: Haisu: Hierarchically supervised nonlinear dimensionality reduction
Source: PLoS Comput Biol. 2022 Jul 21;18(7):e1010351. doi: 10.1371/journal.pcbi.1010351 (PMC9345488; doi:10.1371/journal.pcbi.1010351)
Supplement: Attachment — Submitted filename: ResponseToReviewers.docx [file pcbi.1010351.s002.docx]

**Manuscript ID:** *PCOMPBIOL-D-21-02208*
*Title:* *Haisu: Hierarchical Supervised Nonlinear Dimensionality Reduction*

**Editors**

Dear Dr Cobanoglu,

Thank you very much for submitting your manuscript "Haisu: Hierarchically Supervised Nonlinear Dimensionality Reduction" for consideration at PLOS Computational Biology.

As with all papers reviewed by the journal, your manuscript was reviewed by members of the editorial board and by several independent reviewers. In light of the reviews (below this email), we would like to invite the resubmission of a significantly-revised version that takes into account the reviewers' comments.

We cannot make any decision about publication until we have seen the revised manuscript and your response to the reviewers' comments. Your revised manuscript is also likely to be sent to reviewers for further evaluation.

Thank you again for your submission. We hope that our editorial process has been constructive so far, and we welcome your feedback at any time. Please don't hesitate to contact us if you have any questions or comments.

Sincerely,

Dina Schneidman
Software Editor
PLOS Computational Biology

We appreciate the time and consideration. We have addressed comments from each reviewer with detailed notes and changes. We have changed the structure of the paper, as requested by the reviewers. We have drastically modified the Design and Implementation *(previously Methods)* section, adding a clearer explanation of Haisu’s effect on a NLDR approach. With this, we have added additional user specifiable parameters for better control of the resulting embedding and determination of inter-class and intra-class distances for connected and disconnected components in the hierarchy graph including the built-in support of edge weights. Finally, we have added a new figure (Fig. 1) for easier understanding of the Haisu parameters and exploration of Haisu with a simple random dataset.

**Reviewer 1**

Reviewer #1:

The authors proposed an interesting approach for conducting nonlinear dimensionality reduction by incorporating hierarchical label and tested its performance on three single-cell
RNA-sequencing datasets (embedded with commonly-used packages like t-SNE, UMAP, and PHATE). The concept of the proposed technique is generally straightforward and well described like investigating the embedding effect of TA1 cells without labels, explaining how to determine hierarchical distancing factor(str), and the main findings are validated with multiple strategies.

The authors modify the premier visualization-centric techniques in this field, which has a
significant merit and may potentially become a popular tool, if fully validated, for handling an overwhelming and rapidly-increasing amount of single-cell sequencing data. However, I do have some concerns before considering publication:

Thank you for the review and comments. We have aimed to address all concerns and clarify each point in the following responses.

1. If the hierarchical label information is inserted into the step of “feature selection”, will the pipeline generate same or similar results? Moreover, if the user-defined (a prior called by the authors in the manuscript) hierarchical information is applied for dimensionality reduction, would it lead some leakage of labeling info for subsequent unsupervised clustering?

Thank for the inquiries, as a means of NLDR dimensionality reduction for visualization, Haisu can be employed in any given pipeline but is independent of feature selection and unsupervised clustering in high dimensional space.

Feature selection occurs prior to visualization using a NLDR method which is the sole application of Haisu as presented. Thus, a perturbation or influence of any kind during this step, including one using hierarchical label information, is not relevant to Haisu.

Likewise, we argue that unsupervised clustering and visualization are fundamentally disparate. Haisu is designed exclusively for visualization using dimensionality reduction, thus we cannot relate Haisu or its effects to clustering as an unsupervised learning problem. As applied in typical practice, unsupervised clustering takes place prior to the visualization step.

To examine the use of dimensionality reduction alongside visualization we highlight the commonly termed problem: the ‘curse of dimensionality,’ where critical information can be concealed by irrelevant attributes when observing high-dimensional data^1^. Visualization using NLDR suffers from the same problem: visualization using SNE and t-SNE, for example, can sacrifice density information because of its priority to preserve local neighborhoods^2^. UMAP constructs a simplicial complex while enforcing a local-connectivity constraint to better represent global topology but is nonetheless still plagued by the curse of dimensionality reduction especially for use in 2D visualization. An example of this observation in the field of single-cell RNA sequencing is Shekar *et al.* which presents a classification scheme for interneurons, employing Louvain-Jaccard and Infomap algorithms for clustering separate from their visualization. This is explicitly defined so that “the tSNE representation was only used for visualization and not for defining clusters”^3^. Thus, we argue that the impact of Haisu on unsupervised clustering is relevant given the context of our hierarchical modification on NLDR approaches for visualization.

1.	Zimek, A., Schubert, E. & Kriegel, H.-P. A survey on unsupervised outlier detection in high-dimensional numerical data. *Statistical Analysis and Data Mining: The ASA Data Science Journal* **5**, 363–387 (2012).

2.	Schubert, E. & Gertz, M. Intrinsic t-Stochastic Neighbor Embedding for Visualization and Outlier Detection. in *Similarity Search and Applications* (eds. Beecks, C., Borutta, F., Kröger, P. & Seidl, T.) 188–203 (Springer International Publishing, 2017). doi:10.1007/978-3-319-68474-1_13.

3.	Comprehensive Classification of Retinal Bipolar Neurons by Single-Cell Transcriptomics - ScienceDirect. https://www.sciencedirect.com/science/article/pii/S0092867416310078?via%3Dihub.

We have added more clarification in heading to the Result section, and specify in the Abstract and Introduction explicitly as well to ensure there is no confusion as to the use of Haisu as a visualization method:

*“In summary, Haisu extends existing popular visualization methods to enable a user to incorporate labels known a priori into a visualization, including their hierarchical relationships as defined by a user input graph.” (Abstract).*

*“We focus on the use of dimensionality reduction for visualization of biomedical data.” (Introduction)*

*“Haisu is a generalizable extension to nonlinear dimensionality reduction for visualization that incorporates an input hierarchy to influence a resulting embedding.” (Results).*

2. One of major advantages by performing single-cell sequencing is to break through the current hierarchical understanding of cell differentiation. Here it is applied as a prior knowledge, would it become an obstacle to uncovering new insights?

Thank you, this is an important consideration. We expect the most prevalent use case of Haisu to be in applications where an applied hierarchical prior is desired. If the desired insight is to break away from the supplied hierarchy, then likely the method should not be used. We first inspect the value and importance of hierarchies in the single-cell sequencing domain and how Haisu can be used to highlight these relationships. Second, we stress that Haisu is not limited to single-cell datasets and discuss how hierarchies not related to cell differentiation can be applied effectively *a priori*. Notably, in Fig. 5 we illustrate how our method can accommodate a ‘hybrid,’ semi-supervised style of hierarchical analysis allowing for the presence of labelled and unlabeled datapoints.

We argue that for single-cell sequencing, hierarchies are often immensely valuable and informative, and Haisu can effectively augment these existing priors to better differentiate classes in a visualization. Furthermore, there are multiple domains in biomedicine where strict hierarchical relationships exist and are informative in the analysis of a dataset, on major example being hematopoiesis. In our manuscript we examine the impact of Haisu on three datasets with three distinct types of hierarchies:

1. **Fresh PBMCs:** A hierarchy to differentiate between T cell sub-types based on function. Here, we provide an example of a graph we have supplied to investigate sub-categories of the overlapping labelled T cells in the original embedding. Notably within the “Conventional CD4 T Cell” class, we separate “Effector/Memory” cells from “Naïve T Cells” based on function not cell differentiation. Notably, the authors of this dataset employ hierarchical clustering in their classification (Zheng *et al*)*.*
2. **Embryonic Cardiac Cells:** A hierarchy based on the authors’ supplied hierarchy that is separated by physical proximity of classes, not cell differentiation (Li *et al.*). Some of the classes in this hierarchy include: “Left Atrium,” “Right Atrium,” “Left Ventricle,” and “Right Ventricle” all denoting the location of the sample taken.
3. **Colon Epithelial Cells**: A hierarchy provided by *Smillie* *et al.* based on epithelial differentiation. We highlight our use of the unmodified hierarchy here in the presence of largely overlapping classes in each visualization. This serves as a valuable example where a known hierarchy based on cell differentiation can be used to augment the visualization of an underlying hierarchy.

However, within the constraints of the accompanying NLDR method employed, Haisu is not limited to the single-cell sequencing domain. Some examples where a prior hierarchy can be used to investigate a single-cell dataset in a potentially beneficial manner include but are not limited to:

- Using a hierarchy outside of the domain of cell differentiation to uncover new insights. For example, hierarchy by proximity in the cardiac dataset (Fig. 3), where the prior is defined by the sample location rather than the differentiation hierarchy.
- Exaggerating or modification the known understanding of cell differentiation to investigate potential for subclasses or new classes in the data. For example, in Fig. 5, where the removal of TA 1 label from the data spreads the embedding of the unlabeled data to other hierarchically influenced clusters.

***Fig. 5 (t-SNE):*** *Unlabeled TA 1 cells (grey) cluster near hierarchically influenced Cycling TA cells (blue) at A’ and TA2 cells (pink) at B’.*

3. It seems that the selection criteria of hierarchy relationships in three datasets are different, will this treatment cause some biased outcomes? And, is there any principled guidance to help determine the most appropriate hierarchy labels for independent datasets?

We have added a section to better guide the construction and choice of input graph when using Haisu: “Graph Construction for Haisu” in *Design and Implementation*:

*“We construct a “path-pairwise” matrix* $H=\left( h_{ij} \right)$*, where* $h_{ij}=\theta_{ij}\cdot d_{ij}$*, that modifies the raw distance matrix* $D=\left( d_{ij} \right)$*. H is normalized between [0,1] which has implications on the choice of hierarchy graph regarding inter-class distance, intra-class distance, and the effect of explicit or implicit edge weights on the resulting matrix H…”*

Using different types of hierarchy relationships is intentional to demonstrate that Haisu can be employed with a high level of user configurability across domains and desired hierarchies. In this manner, the reviewer is entirely correct that this will generate biased outcomes, and this is intended. As a configurable visualization method, Haisu should *by design* bias the resulting embedding based on the input hierarchy. Thus, the “most appropriate” hierarchy label set or graph for a dataset depends on the user and their intention. The method adapts to multiple, independent hierarchies for the same dataset.

Line 339 on Page 16: *“However, the effect of the hierarchy can easily be modified for a desired effect.”*How to help users to walk around this trap?

In context, the effect of the hierarchy here refers to the weights of hierarchy graph edges and classes/labels. Thank you, we have clarified this in the passage:

*“However, the effect of the hierarchy can easily be modified for a desired visualization. To better express a hierarchy in an embedding, one could introduce a diminishing factor such that nodes deeper into the graph have a reduced influence as opposed to the base classes” (Availability and Future Directions).*

We help users support multiple hierarchy configurations to better suit different applications by various means:

1. Users can modulate the global weight of edges, via the strength (*str*), or hierarchical distancing factor.
2. Users can modulate the local weight of edges by providing input graph edge weights.
3. Users can influence intra-class distance through modification of *self-distance* and use of *avoid-self*, controlling the path distance from a node to itself.
4. Users can influence the local assignment weight of datapoints especially when class certainty is not 100%. We discuss this extension using probability-based weights per feature.
5. Haisu can be extended to support a different objective function, supporting directional edges and/or weighted edges to prevent the undesired bias of graph label distance.

We don’t regard the configurability of Haisu as a trap for the user. “Desired effect” more generally as the appearance of the embedding can follow any objective function as the problem and user requires it. For example, in Figure S3, we demonstrate an example heuristic for achieving a desired *cluster overlap*. This also can help guide the choice of *str* independent of the NLDR hyperparameters. Another objective function or “desired effect” could be to maximize distance between unrelated labels.

***Fig. S3:*** *We demonstrate a heuristic for aiding in the choice of hierarchical distancing factor (str).*

To alleviate unintended, destructive modification of the hierarchically modified NLDR techniques discussed, we have expanded *Design and Implementation*:

*“At high values of str and/or low values of self-distance, Haisu can destructively modify the integrity of the NLDR so that intra-class distances are drastically different after application of H. For all the specified NLDR methods in this section, asymmetric similarity calculation is critical to downstream analysis and case 1 will have the following effect:* $\hat{s}_{j|i}= 1 | \theta_{ij}= 0$*. This will also affect the cost function*$\hat{C}_{ij}=0 | i^{'}=j'$ *so that neither low nor high-dimensional coordinates are employed between points of the same class, hence our limitation of str < 1 (Fig. 2-5).” (Design and Implementation).*

4. Line 354 on Page 17: *“We found its effect to be highly dependent on the characteristics of the dataset and the size/structure of the input hierarchy.”* I wonder what aspect of the characteristics of a new dataset will affect the size/structure of the input hierarchy and how?

Thank you for highlighting this point, we have added additional information and a new section in *Design and Implementation* to better provide insight into what influences the effect of Haisu due to a user’s choice of parameters (including *str* as referred to in this passage) and the user’s choice of structure/weights for the input hierarchy.

However, we argue that characteristics of a new dataset do not affect the size/structure of the hierarchy which is by design provided *a priori*. A dataset and the size/structure of an input hierarchy are fundamentally independent because the user can supply any prior irrespective of its implicit or explicit relation to the data. Rather, we aim to highlight what aspects of a dataset, NLDR approach, and choice of additional parameters affect the user’s choice of *str*.

The quoted passage refers the strength / hierarchical distancing factor $str$ which does have distinct contributors toward its scale of effect. Ultimately, distances of datapoints in the raw embedding for a given NLDR technique (without hierarchical influence) and how the modified distance matrix is employed per technique have the most influence on the scale and choice of strength factor *str*. We stress that visualization is in general subjective by nature, though some factors that contribute to a value of *str* that may be visually preferrable for reducing crowding and over exaggeration of the hierarchy in an embedding include:

- High dimensional distances between datapoints
- Choice of NLDR approach
- Choice of hyperparameters for the NLDR approach (ex. *Perplexity*)
- Construction and size of the hierarchy graph *G*, as supplied to Haisu
- Contribution of edge weights in *G*
- Per-datapoint class probability weighting
- Specification of inter-class distance via *self-distance* and the set *avoid-self*
- A presence of disconnected components in *G* and choice of relevant parameters

To this effect, we have better outlined how the choice of NLDR hyper parameters and Haisu parameters can influence the final graph in *Design and Implementation*.

*“To minimize potential crowding in the embedding and better represent the original NLDR method, at higher values of str Haisu will benefit from a lower user-provided perplexity, n_neighbors, and knn value which estimate or determine the number of nearest neighbors during the calculation of asymmetric similarities* $\hat{p}_{j|i}$ *for t-SNE, UMAP, and PHATE respectively” (Design and Implementation).*

*“UMAP allows for a “minimum distance” parameter that determines the minimum distance of points in low-dimensional space. Because Haisu reduces* $\hat{p}_{ij}$ *relative to* $p_{ij}$*, at higher values of str, one may want to increase “minimum distance” if a visualization similar to the unmodified embedding or preservation of global structure is desired.”*

*“The degree of Haisu’s modification H is even greater than in t-SNE due to the exponential decay factor. Thus, due to the closer high-dimensional distances, Haisu will benefit from a lower user-provided knn parameter and lower decay factor to prevent potential crowding in the embedding.”*

Additionally, we have expanded the *Design and Implementation* section to better describe the effect of a user-defined hierarchy on the local and global structure preservation of an embedding. To this effect, we have added additional parameters for the user to better control how and which labels/classes interact.

*“…We also include several parameters to drive the effect of the hierarchy graph* G: disconnected-distance, self-distance, *and an array of unique labels* avoid-self*. Finally, users can specify the explicit weights of edges prior to normalization of H.”(Design and Implementation)*

***Fig. 1:*** ***Users*** ***can control the effect of an input hierarchy on the resulting embedding.*** *We demonstrate the effect of Haisu as applied to t-SNE, UMAP, and PHATE with an input graph on a set of random points…*

*“Beyond the use of str, one can reduce the effect of Haisu on an NLDR technique by increasing self-distance above 0 so that* $\forall x_{i},x_{j}\in X\to\theta_{ij}>0$ *within a connected component of G. Thus, in situations where it is more desirable to preserve characteristics of the NLDR technique or reduce clustering, one should consider increasing self-distance and adding respective classes to avoid-self. In Fig. 1 we demonstrate the effect of modulating this value on a set of random points…” (Design and Implementation)*

5. Regarding the definition of the distance modification function in Results (Line 109-112 on Page 7), it is given as follows: Is it in effect equivalent to the equation (Line 421-424 on Page 18)

Thank you for this consideration, we have restructured the definition of $\theta_{ij}$ and related discussion. The equation for $\theta_{ij}$ was supplied on Page 7, and its application to the pairwise probability calculation of t-SNE was listed on Page 18.

For better clarity, we have improved the writing and moved detailed explanation of the method to *Design and Implementation*, with a better explanation of how Haisu affects a given NLDR approach at the beginning of Results.

*“Haisu is a generalizable extension to nonlinear dimensionality reduction for visualization that incorporates an input hierarchy to influence a resulting embedding. We achieve this through a hierarchical distancing factor* $\theta$ *which directly modifies an existing pairwise distance function* $d$ *used in the calculation of asymmetric similarities for two points in high-dimensional space prior to embedding. Specifically, Haisu’s modification is* $\theta_{ij}\cdot d(x_{i},x_{j})$ *for a high dimensional dataset* $\mathcal{X=}\{x_{1},x_{2},\ldots,x_{N}\}$*” (Results).*

*“Our algorithm introduces* $\theta_{ij}$ *as a direct modification to the distance function based on the input label hierarchy where* ${0\leq\theta}_{ij}<1$*. As applied to an existing nonlinear dimensionality technique, Haisu produces a modified pairwise distance function…” (Design and Implementation).*

**Reviewer 2**

Reviewer #2: Real world data often lies in low-dimensional manifolds embedded in high-dimensional spaces, with high codimensions (manifold hypothesis). It is generally a hard task to obtain projections from high-dimensional spaces to visualizable spaces, e.g. of dimension 2 or 3, such that the properties that characterize the low-dimensional embedding are preserved. There are several techniques employed for dimensionality reduction, typically divided into linear and nonlinear classes. The latter class includes t-SNE, UMAP and PHASE.

In the article under consideration, the authors propose a method that modifies the distances used in t-SNE, UMAP and PHASE by means of user-defined hierarchies. This method results in a modification of the projection that reduces the dimensionality, parametrized by a strength factor decided by the user. The main focus of this approach is on biomedical data, and the authors provide experiments on single-cell RNA sequencing datasets.

The article is of value, and it should be considered for publication in the journal PLOS Computational Biology. However, the article currently needs significant revisions, as described below. The main issue lies in the fact that the method is overall not well explained, and some natural questions regarding the relation between t-SNE, UMAP, PHASE and the corresponding modified versions proposed in this article are not properly discussed. Also, the organization of the article does not seem to be ideal, and it would be greatly beneficial to restructure it.

Thank you for your suggestions, we have substantially revised both the general structure of the paper and specifically the descriptions of the method as you requested. More details are included in the following responses.

-- The subsection named “Hierarchically Supervised NLDR” is not working at the moment. There are several issues with the exposition. For instance, the setting is not well explained, and it can only be guessed by the reader. There is a high dimensional input dataset, but it seems that the input should also consist of a hirarchy graph. This is mentioned at the end of the first paragraph (line 100), but it should be manifest from the beginning. It is not clear what “variable distance function” means here. It seems that variable here means “not constant”. However, distances cannot be constant, as this would violate the triangular axiom. So, one might deduce that variable here means something else, and the authors are trying to convey that the distance satisfies this property of being variable. Also, the term “distance modification” appearing in line 98 is also unclear at this point. It is clarified (implicitly) towards the end of the manuscript, in the Methods section, but it should not be left unclear for so long. The graph “G” seems to be the hirarchy graph mentioned above. This should be explicitly stated.

Thank you for this clarification. As requested, we have explicitly specified *G* as the hierarchy graph and a second input to the algorithm to aid in the exposition of the method. We have removed the use of “variable” meaning “modular” (referring to the final application) as it is not needed and confusing in the declaration of the method. Finally, we have removed the term “distance modification” in favor of an explicit meaning.

*“Given a high dimensional input* $\mathcal{X=}\{x_{1},x_{2},\ldots,x_{N}\}$*, the asymmetric similarity* $s_{j|i}$ *between* $x_{i},x_{j}$ *is commonly determined in-part by employing a distance function* $d\left( x_{i},x_{j} \right)$*. Furthermore, given an undirected (not necessarily connected) input hierarchy graph* $G=(V_{G}, E_{G})$ *with labels assigned to each node, we process input high-dimensional points* $x_{i}, x_{j}$ *with labels* $i^{'},j^{'}$ *respectively… Our algorithm introduces* $\theta_{ij}$ *as a direct modification to the distance function based on the input label hierarchy where* ${0\leq\theta}_{ij}<1$*” (Results).*

Similarly, the term ``strength’' in line 106 does not correspond to any canonical notion in the computational sciences. The meaning can be deduced afterwards from line 110, but again, this is not helping the reader. More generally, in this first subsection, the relation between theta_{ij} and the methodology is completely unclear. It is only understood that it modifies the distance, but it is not clear how until the last section of the article. This subsection could be merged with the last section for clarity, and rewritten. If the authors prefer to discuss the technicalities after showing the results, this could be done too. A brief general description without using equations could be added as the first subsection of the article, and a detailed account (current first subsection and last section merged together and better exposed) could appear at the end. The current situation lies somehow in between.

Per suggestion, we have migrated more detailed explanation of equations to *Design and Implementation* and added a brief general description of the approach prior to example application.

*“Haisu is a generalizable extension to nonlinear dimensionality reduction for visualization that incorporates an input hierarchy to influence a resulting embedding. We achieve this through a hierarchical distancing factor* $\theta$ *which directly modifies an existing pairwise distance function* $d$ *used in the calculation of asymmetric similarities for two points in high-dimensional space prior to embedding. Specifically, Haisu’s modification is* $\theta_{ij}\cdot d(x_{i},x_{j})$ *for a high dimensional dataset* $\mathcal{X=}\{x_{1},x_{2},\ldots,x_{N}\}$*. A parameter* $str\in[0,1)$ *can be supplied to modify the “strength” of the hierarchy’s effect on the distance function. When* $str=0$*, the original distance function is preserved and the resulting embedding is unmodified relative to the raw NLDR technique. At* $str>0$ *,* $d$ *is multiplied by an interpolated factor determined by normalized path distances in the input hierarchy and the value of* $str$*… Haisu can be immediately applied to any NLDR technique compatible with the precomputed “path-pairwise” distance matrix* $H=\left( \theta_{ij} \right)$*, where* $H$ *is multiplied by the unmodified distance matrix* $D=\left( d_{ij} \right)$*” (Results)*

As requested, we have clarified the relation between $\theta_{ij}$ and the methodology, establishing a better explanation of the term “strength” as well. In doing so, we have migrated explanation of $\theta_{ij}$ from *Design and Implementation* to Results as it relates to a NLDR technique to better facilitate the introduction of the method as suggested. This is achieved by specifying *d’* and how $\theta_{ij}$ modifies a pairwise distance function explicitly instead of implicitly as before.

*“Our algorithm introduces* $\theta_{ij}$ *as a direct modification to the distance function based on the input label hierarchy where* ${0\leq\theta}_{ij}<1$*. As applied to an existing nonlinear dimensionality technique…”*

*“To calculate* $\theta_{ij}$*, we find the shortest path distance of two undirected nodes* $\overset{⃑}{j^{'}i^{'}}$ *in G. Define maxdist as the maximum length shortest path in G; in other words, maxdist is defined as max(S) where S is the set of possible shortest paths in G. Finally, let* $str\in[0,1)$ *denote a parameter to modulate the “strength” of the hierarchical distancing factor* $\theta_{ij}$ *as applied to the pairwise-distance function* $d\left( x_{i}, x_{j} \right)$*. At lower values of* $str$*, the distance* $d\left( x_{i},x_{j} \right)$ *is preserved, and at higher values, distances are penalized based on the corresponding hierarchy graph” (Design and Implementation).*

Regarding the use of “strength” we have also fixed an inconsistency in the following sentence: should be *str* not $\theta_{ij}$.

*“At lower values of* $str$*, the distance* $d\left( x_{i},x_{j} \right)$ *is preserved, and at higher values, distances are penalized based on the corresponding hierarchy graph” (Design and Implementation).*

In line 115, the use of s.t. (such that), does not seem to follow the traditional practice in mathematics. It seems rather that the authors meant to say “so that”. But s.t. means “such that”, which is also indicated by a vertical bar in sentences using the formalism of mathematical logic. In line 121, it seems that the quantity “m” could be obtained via min, max or other types of aggregation. The authors say that the probability could be taken to be min, max or other types of aggregation. This does not seem to be correct. Also, “m” seems to depend on i,j, so this dependence should be explicitly shown in the notation because “m” is not a parameter common to all x_i, x_j.

Thank you, we have fixed the notation of “*s.t.”* and “*m”* as suggested in *Design and Implementation*.

*“If a label does not exist for* $x_{i}$ *or labels* $i^{'},j^{'}$ *are in different connected components, then* $\overset{⃑}{j^{'}i^{'}}=maxdist$ *and* $\theta_{ij}=1$*”*

$$\theta_{ij}= \left( 1-m_{ij}\cdot str \right)+ \frac{m_{ij}\cdot str\left( \overset{⃑}{j^{'}i^{'}}- 1 \right)}{maxdist - 1}$$

Regarding the modularity of the aggregation function, we have removed the passage stating that the probability could employ a different type of aggregation, as suggested, in favor of representing the method as implemented.

-- The Benchmark subsection is overall well written. However, the tests that have been to evaluate the ability of this method to preserve the structure of the embeddings generated by t-SNE, UMAP, PHASE do not seem to be complete. The results regarding the relative positioning of points found here (line 269 on) are encouraging, but the aforementioned methods were motivated by deep analytical, geometrical and topological reasons which are not considered here. It would be reasonable to expect that increasing the strength coefficient the perturbation on the distances increasingly alters the geometric structure until the latter is lost. If on the one hand it would be unreasonable to expect a full understanding of these mechanisms in this article, it is on the other hand good to provide at least a perspective on this matter, as it can be an important factor in the fine tuning of the parameters for specific applications.

Thank you for this feedback. We have expanded the section “The Effect of Haisu on t-SNE, UMAP, & PHATE” to better analyze the deeper mathematical ideas underlying each approach. We have also added a section in *Design and Implementation* to better evaluate the effect of *str* and its effect on the local and global topology of a given NLDR technique. Furthermore, we have added additional parameters for the user to better control how and which labels/classes interact.

We have expanded descriptions in *Design and Implementation* of the impact of Haisu on the local and global structure preservation of each NLDR method’s embedding:

*“T-SNE aims to minimize the separation between the data points in the original high-dimensional space and the low-dimensional projection space. As such, it makes no topological guarantees except for the implication that points close in the high-dimensional space will likewise be close to each other in the projection. Thus, the neighborhood matrix is of fundamental importance since the construction of the final embedding is focused on preserving neighborhoods in the low-dimensional projection. Using Haisu, we perturb the high-dimensional space to impart path distances in the hierarchical label graph onto the neighborhood relationships by reducing distances in high-dimensional space. The higher the global ‘strength’ value, local edge weight, and label probabilities assigned to pairs, the stronger this effect. Datapoints with closer labels in the hierarchy are prioritized during the minimization of Kullback-Leibler divergence due to the reduced pairwise similarity and thereby have a higher propensity to be identified as neighbors. Consequently t-SNE is modified by Haisu in such a way that the low dimensional projection reflects the input hierarchy.”*

*“UMAP is topologically motivated in that the method aims to identify a simplicial set covering the dataset that recovers the structure in the high-dimensional space and seeks to reproduce it in the projection. Haisu will affect the determination of the final embedding by determining the degree to which the topological structure of the manifold is preserved via the influence of the input hierarchy. Higher values of str, edge weights, and label probabilities for pairs* $x_{i}, x_{j}$ *will increase the propensity of similar classes in the hierarchy to exist as k nearest neighbors in the manifold. This can affect the local-connectivity constraint* ${\theta_{ij}\cdot n}_{i}$*, and directly affects the directed edges in the weighted k-neighbor graph via* $\hat{s}_{j|i}$ *and the resulting probability that at least one of the two edges from* $x_{i}$ *to* $x_{j}$ *or* $x_{j}$ *to* $x_{i}$ *exists via* $\hat{p}_{ij}.$ *Thus when using HAISU, UMAP is modified in a manner that the topological representation of the high-dimensional data and resulting embedding reflects the input hierarchy.”*

*“PHATE models a diffusion process aiming denoise the data and represent global and local information, using multidimensional scaling (MDS) to project the information to lower-dimensional space. Like t-SNE, PHATE does not make any topological guarantees but encodes local data using local similarities and global data via potential distance via diffusion. Local similarities between* $x_{i}$ *and* $x_{j}$ *using Euclidean distances and the Markov-normalized affinity matrix using the* $\alpha$*-decaying kernel function* $\hat{s}_{j|i}$ *are influenced so that datapoints with closer labels in the hierarchy will have greater local similarities and higher initial random walk probabilities as a result. Potential distances employ the local similarities to determine t-step walk probabilities, hence, in the normalization of local affinities Haisu will also affect the global determination of PHATE as it contributes to the M-divergence. In this manner, PHATE’s sensitivity to small distances in the probability distribution corresponding to larger global structures is especially impacted by Haisu through the decay factor* $\alpha$*. At high values of str, PHATE will often produce an embedding with branches that resemble the input hierarchy to a greater extent than UMAP or t-SNE given self-distance is sufficiently small or zero.”*

We have added a section to better guide the construction and choice of input graph when using Haisu: “Graph Construction for Haisu” in *Design and Implementation*:

*“We construct a “path-pairwise” matrix* $H=\left( h_{ij} \right)$*, where* $h_{ij}=\theta_{ij}\cdot d_{ij}$*, that modifies the raw distance matrix* $D=\left( d_{ij} \right)$*. H is normalized between [0,1] which has implications on the choice of hierarchy graph regarding inter-class distance, intra-class distance, and the effect of explicit or implicit edge weights on the resulting matrix H” (Design and Implementation).*

***Fig. 1:*** ***Users*** ***can control the effect of an input hierarchy on the resulting embedding.*** *We demonstrate the effect of Haisu as applied to t-SNE, UMAP, and PHATE with an input graph on a set of random points…*

*“**Beyond the use of* $str$*, one can reduce the effect of Haisu on an NLDR technique by increasing self-distance above 0 so that* $\forall x_{i},x_{j}\in X\to\theta_{ij}>0$ *within a connected component of G. Thus, in situations where it is more desirable to preserve characteristics of the NLDR technique or reduce clustering, one should consider increasing self-distance and adding respective classes to avoid-self. In Fig. 1 we demonstrate the effect of modulating this value on a set of random points” (Design and Implementation).*

For the Benchmarks subsection, we have also added how these new parameters are employed for additional clarity.

*“…We also include several parameters to drive the effect of the hierarchy graph G: disconnected-distance, self-distance, and an array of unique labels avoid-self. Finally, users can specify the explicit weights of edges prior to normalization of H.”(* *Design and Implementation)*

*“To alleviate intra-class clustering of points with the “Dummy” label, we specify Haisu construction parameter self-distance as 1 and include “Dummy” in the set of labels avoid-self as described in Design and Implementation. We also set disconnected-distance to 1 which encourages graph-disconnected TA 1 cells in the embedding to instead cluster near their next most similar class. In combination, these parameters ensure* $\theta_{ij}=1$ *when the labels of* $x_{i}$ *or* $x_{j}$ *are “Dummy” which maintains high-dimensional distances from the raw NLDR approach.” (Results)*

We have better outlined how the choice of NLDR hyper parameters and Haisu parameters can influence the final graph in *Design and Implementation*.

*“To minimize potential crowding in the embedding and better represent the original NLDR method, at higher values of str Haisu will benefit from a lower user-provided perplexity, n_neighbors, and knn value which estimate or determine the number of nearest neighbors during the calculation of asymmetric similarities* $\hat{p}_{j|i}$ *for t-SNE, UMAP, and PHATE respectively” (Design and Implementation).*

*“UMAP allows for a “minimum distance” parameter that determines the minimum distance of points in low-dimensional space. Because Haisu reduces* $\hat{p}_{ij}$ *relative to* $p_{ij}$*, at higher values of str, one may want to increase “minimum distance” if a visualization similar to the unmodified embedding or preservation of global structure is desired.”*

*“The degree of Haisu’s modification H is even greater than in t-SNE due to the exponential decay factor. Thus, due to the closer high-dimensional distances, Haisu will benefit from a lower user-provided knn parameter and lower decay factor to prevent potential crowding in the embedding.”*

-- There is an issue with considering the ``Effect of HAISU on t-SNE, UMAP, and PHATE’’, as in the last section of the article. Until now, no general understanding of the method has been provided to the reader. So, as far as the reader knows, up to now, HAISU is a method that alters t-SNE, UMAP, and PHATE to produce better embeddings. In fact, the only explanation regarding the way the coefficients theta_{ij} descibed at the beginning of the article influence the projection is provided in this section. In other words, this is the actual definition of the method, not the analysis of the effect of the method on t-SNE, UMAP, and PHATE. The method is not considered in generality, even though some experiments on PCA are also provided to
compare to linear methods. One could only guess how the definition goes in the case of PCA. From the codes one might get a clearer understanding of how to apply HAISU in general, but it is not ideal.

In accordance with this and previous feedback, we have restructured the definition of Haisu and use of $\theta_{ij}$, describing the explicit effect *in Design and Implementation* and providing an overview in Results. Furthermore, this better foundation introduces the technique more generally as it can be applied to more than the popular methods presented in the manuscript.

Regarding PCA: We have also added clarification as to the use of the pairwise distance matrix, its application to any compatible NLDR implementation, and how PCA is applied.

*“Haisu can be immediately applied to any NLDR technique compatible with the precomputed “path-pairwise” distance matrix* $H=\left( \theta_{ij} \right)$*, where* $H$ *is multiplied by the unmodified distance matrix* $D=\left( d_{ij} \right)$*. We illustrate the effect of Haisu on PHATE, t-SNE, and UMAP at multiple strength factors, and include PCA (executed on* $H$*) to express more simply how Haisu modifies the pairwise distance matrix” (Results).*

-- The right hand sides of the equations in lines 419 and 422 seem identical. It seems that there is a factor of theta_{ij} in line 419 that should not appear.

We greatly appreciate the correction and have removed the typo in the definition of $s_{j|i}$ for t-SNE in *Design and Implementation*:

$$s_{j|i}= exp(-d\left( x_{i}, x_{j} \right)^{2}/ 2 \sigma_{i}^{2})$$

________________________________

Have the authors made all data and (if applicable) computational code
underlying the findings in their manuscript fully available?
The PLOS Data policy requires authors to make all data and code
underlying the findings described in their manuscript fully available
without restriction, with rare exception (please refer to the Data
Availability Statement in the manuscript PDF file). The data and code
should be provided as part of the manuscript or its supporting
information, or deposited to a public repository. For example, in
addition to summary statistics, the data points behind means, medians
and variance measures should be available. If there are restrictions
on publicly sharing data or code —e.g. participant privacy or use of
data from a third party—those must be specified.

Reviewer #1: Yes

Reviewer #2: No:

We have included an updated git repository will full source code updated with the new parameters: https://github.com/Cobanoglu-Lab/Haisu

________________________________

PLOS authors have the option to publish the peer review history of
their article (what does this mean?). If published, this will include
your full peer review and any attached files.


If you choose “no”, your identity will remain anonymous but your
review may still be made public.


Do you want your identity to be public for this peer review? For
information about this choice, including consent withdrawal, please
see our Privacy Policy.

Reviewer #1: Yes: Zheng Wang

Reviewer #2: No

________________________________

Figure Files:

While revising your submission, please upload your figure files to the Preflight Analysis and Conversion Engine (PACE) digital diagnostic tool, [https://pacev2.apexcovantage.com](https://pacev2.apexcovantage.com/). PACE helps ensure that figures meet PLOS requirements. To use PACE, you must first register as a
user. Then, login and navigate to the UPLOAD tab, where you will find detailed instructions on how to use the tool. If you encounter any issues or have any questions when using PACE, please email us at figures@plos.org.

Data Requirements:

Please note that, as a condition of publication, PLOS' data policy requires that you make available all data used to draw the conclusions outlined in your manuscript. Data must be deposited in an appropriate repository, included within the body of the manuscript, or uploaded as supporting information. This includes all numerical values that were used to generate graphs, histograms etc.. For an example in PLOS Biology see here:
<http://www.plosbiology.org/article/info%3Adoi%2F10.1371%2Fjournal.pbio.1001908#s5>.

Reproducibility:

To enhance the reproducibility of your results, we recommend that you deposit your laboratory protocols in protocols.io, where a protocol can be assigned its own identifier (DOI) such that it can be cited independently in the future. Additionally, PLOS ONE offers an option to publish peer-reviewed clinical study protocols. Read more information on sharing protocols at [https://plos.org/protocols?utm_medium=editorial email&utm_source=authorletters&utm_campaign=protocols](https://plos.org/protocols?utm_medium=editorial%20%20email&utm_source=authorletters&utm_campaign=protocols)
